# Potential applications of *Pseudomonas* sp. (strain CPSB21) to ameliorate Cr^6+^ stress and phytoremediation of tannery effluent contaminated agricultural soils

**DOI:** 10.1038/s41598-018-23322-5

**Published:** 2018-03-20

**Authors:** Pratishtha Gupta, Rupa Rani, Avantika Chandra, Vipin Kumar

**Affiliations:** Department of Environmental Science and Engineering, Indian Institute of Technology (ISM), Dhanbad, 826 004 Jharkhand India

## Abstract

Contamination of agricultural soil with heavy metals has become a serious concern worldwide. In the present study, Cr^6+^ resistant plant growth promoting *Pseudomonas* sp. (strain CPSB21) was isolated from the tannery effluent contaminated agricultural soils and evaluated for the plant growth promoting activities, oxidative stress tolerance, and Cr^6+^ bioremediation. Assessment of different plant growth promotion traits, such as phosphate solubilization, indole-3-acetic acid production, siderophores, ammonia and hydrogen cyanide production, revealed that the strain CPSB21 served as an efficient plant growth promoter under laboratory conditions. A pot experiment was performed using sunflower (*Helianthus annuus* L.) and tomato (*Solanum lycopersicum* L.) as a test crop. Cr^6+^ toxicity reduced plant growth, pigment content, N and P uptake, and Fe accumulation. However, inoculation of strain CPSB21 alleviated the Cr^6+^ toxicity and enhanced the plant growth parameters and nutrient uptake. Moreover, Cr toxicity had varied response on oxidative stress tolerance at graded Cr^6+^ concentration on both plants. An increase in superoxide dismutase (SOD) and catalase (CAT) activity and reduction in malonialdehyde (MDA) was observed on inoculation of strain CPSB21. Additionally, inoculation of CPSB21 enhanced the uptake of Cr^6+^ in sunflower plant, while no substantial enhancement was observed on inoculation in tomato plant.

## Introduction

Chromium (Cr) is the second most abundant metal which enters into the agricultural ecosystem by the application of wastes containing Cr. Cr is widely used in leather tanning, metal finishing, chromate preparation, wood preservation, and alloy preparation due to its properties such as resistance to corrosion, temperature, wear, and decay^1^.

The region Jajmau, Kanpur, India presents about 400 tannery industries^2^. For more than last two decades the treated effluent with high metal concentration is extensively used in the irrigation of ~2100 acres of agricultural land in this region^3^. The government supplies treated effluent and sewage in the area for irrigation purpose as stated in a report published in Down to Earth^4^. The report also conceals that the chromium in the area has now entered into the food chain. Moreover, the production of wheat, paddy, and barseem has reduced to 50%, while flower yield has dropped to 60% due to the use of contaminated water for irrigation in the area. Furthermore, report also highlights the study conducted by National Botanical Research Institute, Lucknow, which ascertains high Cr level in the agricultural produce of the affected villages in Jajmau^4^.

The high Cr concentration promotes negative effects on the plants causing disruption in photosynthetic processes, root hair formation, nitrogen assimilation, electron transport chain, and cell wall metabolism, thus leading to reduced plant biomass production^5^. In addition to these, the higher concentration of Cr cause oxidative stress that results in the production of reactive oxygen species (ROS) causing an increase in membrane permeability due to MDA formation/lipid peroxidation^6^. Under such conditions, plants regulate their metabolism by initiating biochemical changes to protect them from the stress-induced oxidative injury^7,8^.

Remediation of contaminated sites via conventional methods such as land-filling, soil washing, electrokinetic remediation, and excavation are costly and high energy consuming^9^. In this regard, an alternative approach “Bioremediation” has gained greater insight for cleanup of polluted sites due to its cost-effective and environmental friendly nature^10^. For an effective remediation, microbes in the metal polluted environment must cope up with the heavy metal stress^11^. The bacterial strains are able to tolerate heavy metal stress by several resistance mechanisms, including complex formation with thiol-containing molecules, active efflux system, immobilization/mobilization of heavy metals, extra or intracellular sequestration, and conversion of highly toxic form of compounds into less toxic^12,13^. Synergistic associations of plants with metal resistant plant growth promoting rhizospheric (PGP) bacteria offers great potential for remediating contaminated soils^14^. PGP bacteria play a crucial role in alleviating the metal toxicity and exert ameliorating effects on plant growth and mineral uptake^15^. These rhizospheric bacteria promote plant growth through mineral phosphate solubilization, nitrogen fixation, indole-3-acetic acid, siderophores, hydrogen cyanide, and ammonia production^16^. These microbes inhabiting the plant rhizosphere are essential to phytoremediation process due to their effects on enhanced biomass production, plant growth, anti-oxidative enzymes, and metal tolerance ability. Application of selected rhizospheric bacteria can simultaneously increase metal phytoavailability, and reduce toxicity, allowing the plant to produce more biomass and accumulate large amount of metals^11^.

The main objective of the present work was to study the Cr^6+^ bioremediation using PGP bacterial isolate. The PGP activities of the bacterial isolate were also examined in a pot scale experiment. The potential Cr^6+^ resistant bacterial strains were isolated from the contaminated agricultural soils and their PGP traits were determined. It was hypothesized that the inoculation of Cr resistant bacteria with PGP activities can enhance the growth of test plants along with increase in phytoextraction potential under stress conditions due to increase in nutrients and mineral uptake in correlation with the induction of anti-oxidative enzyme production.

## Results

### Analysis of soil samples

The physico-chemical characteristics of the soil samples are described in Supplementary Table (SM) 1. Soil pH was slightly alkaline in nature which may be due to the use of effluent containing basic salts in the tanning process for irrigation purposes. The Cr^6+^ concentration in the soil samples was 18–30 mg kg^−1^. The threshold limit of Cr^6+^ in the soil according to the Ministry of Environment/Ministry of Health^17^ and CCME^18^ is 10 and 8 mg kg^−1^, respectively.

### Isolation of Cr-resistant strains

In this study, 44 isolates were screened out as potential Cr^6+^ resistant bacterial strains. Out of 44, six strains showing high tolerance to Cr^6+^ (up to 700 mg L^−1^) were selected for the evaluation of PGP traits.

### PGP traits of the isolates under Cr^6+^ stress

**Figure 1:**
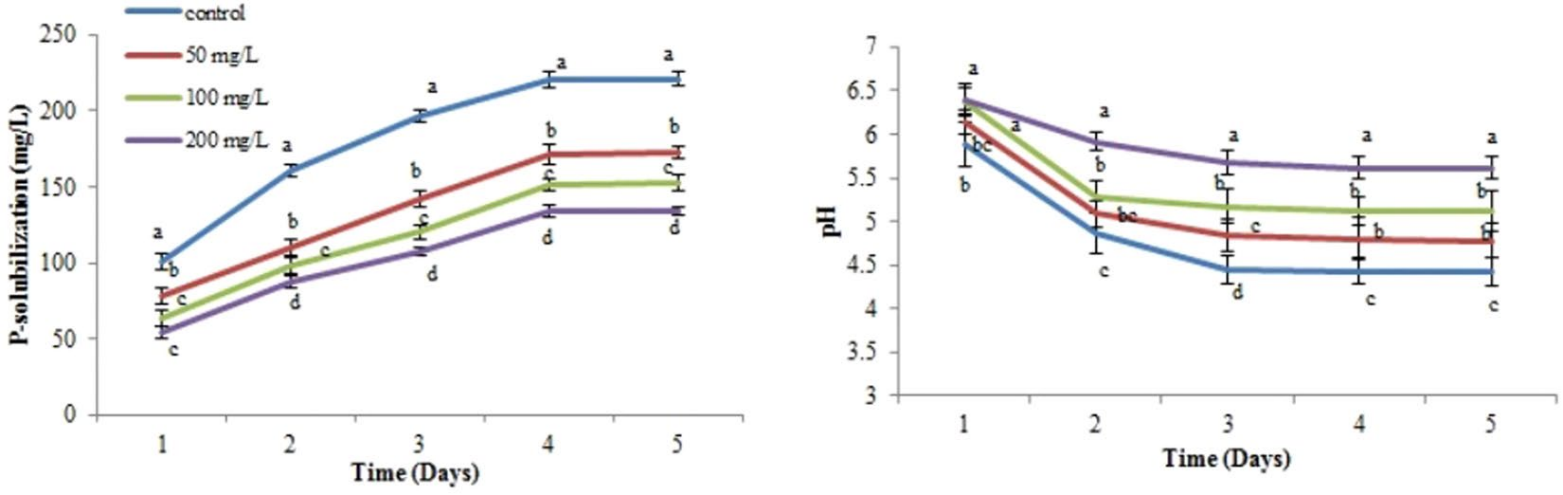
P-solubilization potential of the isolate CPSB21 under Cr^6+^ stress. Values with different alphabets are significantly different from each other according to post hoc Tukey’s HSD (*P* < 0.05). Each value is a mean of three replicates.

The phosphate solubilizing ability of the Cr^6+^ resistant isolates was studied over a period of 120 h by monitoring a drop in pH and available phosphorus in the culture medium. In our study, a substantial decrease in the amount of P solubilized by the isolates was observed to increase in the concentration of Cr^6+^ in the culture media (Table 1). However, maximum solubilization was observed with the isolate CPSB21. The solubilization potential and drop in pH of the isolate CPSB21 is shown in Fig. 1.

**Table 1:** PGP activity by the isolated strains.

Isolates	Cr^6+^ conc. (mg L^−1^)	PGP traits				
		P solubilization (mg L^−1^)	Siderophore zone (cm)	IAA (µg mL^−1^)	HCN	NH_4_
CPSB5	Control	147 ± 6.2ef	1.8	18.2 ± 1.2ghij	+	+
	50	114 ± 4.5ghij	1.5	13.8 ± 1.2jkl	+	+
	100	96 ± 6.2jkl	1.2	10.1 ± 0.7 lm	+	+
	200	78 ± 6.5lmn	1.0	8.3 ± 0.8 m	+	+
CPSB6	Control	194 ± 9.6b	2.6	35.5 ± 2.3a	++	++
	50	152 ± 9.5de	2.2	27.7 ± 2.2 cd	++	++
	100	127 ± 7.2gh	1.9	22.2 ± 1.6efgh	++	++
	200	111 ± 7hijk	1.6	19.6 ± 1.2ghi	++	++
CPSB13	Control	166 ± 6 cd	2.4	25.8 ± 1.4de	++	+
	50	128 ± 6.5gh	2.1	19.9 ± 1.3fghi	++	+
	100	112 ± 6.2hij	1.7	15.7 ± 1.3ijk	+	+
	200	98 ± 6jk	1.5	12.4 ± 1.3klm	+	+
CPSB21	Control	221 ± 9a	3.2	33.9 ± 2.3ab	++	++
	50	172 ± 8.1c	2.8	25.9 ± 1.9de	++	++
	100	152 ± 6.0de	2.4	21.4 ± 1.4fgh	++	++
	200	134 ± 6.9efg	2.1	17.9 ± 1.6hij	++	++
CPSB26	Control	120 ± 8.0ghi	2.3	22.6 ± 2.0efg	++	++
	50	92 ± 7.0klm	2.1	18.2 ± 1.1ghij	++	++
	100	74 ± 6.2mn	1.7	14.3 ± 1.3jkl	++	+
	200	66 ± 4.5n	1.5	11.7 ± 1.5klm	++	+
CPSB41	Control	174 ± 8.1c	3.5	30.8 ± 2.2bc	++	++
	50	131 ± 7.5fgh	3.1	24.1 ± 1.5def	++	++
	100	116 ± 7.5ghij	2.9	19.5 ± 1.2ghi	++	++
	200	104 ± 6.5ijk	2.5	14.2 ± 1.4jkl	++	++

Values with different alphabets are significantly different from each other according to post hoc Tukey’s HSD (*P* < 0.05). Each value is a mean of three replicates.

Production of siderophores by the isolates was qualitatively analyzed on a CAS agar plate. The strains produced halo-zone on the CAS agar plate amended with Cr^6+^ in different concentrations. This shows the potential of the strain for siderophores production under stress conditions. The isolate CPSB41 showed maximum zone formation (Table 1).

The ability of the isolates to produce IAA was determined. Maximum IAA production was obtained with the isolate CPSB6 which decreased with increase in Cr^6+^ concentration (Table 1). Moreover, NH_3_ and HCN production by different isolates was variable under Cr^6+^ stress.

Assessment of different PGP traits revealed isolate CPSB21 as the most efficient strain under stress conditions, hence was selected for the pot trials.

### Metal mobilization potential of CPSB21

**Figure 2:**
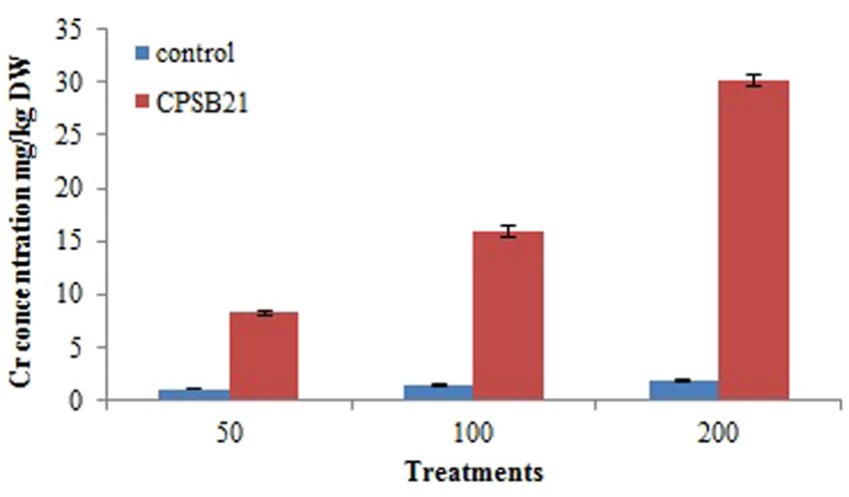
Effect of inoculation with *Pseudomonas* sp. CPSB21 on the mobilization of chromium in soil.

The concentration of water soluble Cr in soil was examined to access the metal mobilization potential of *Pseudomonas* sp. CPSB21 in soil. Compared with control, the inoculation of CPSB21 for seven days increased the concentration of water soluble Cr in soil (Fig. 2), which was 7.5, 11.2, and 16.7- fold higher than those in the control soil for 50, 100, and 200 mg kg^−1^ treatments, respectively.

### Pot trials

The effects of isolate CPSB21 on the growth of sunflower and tomato plants under Cr^6+^ stress is shown in Table 2. Under Cr^6+^ stress, a substantial reduction in shoot length, root length, fresh weight, and dry weight was noticed. However, with CPSB21 inoculation commendable enhancement in all the plant growth parameters was observed in both the plants (Table 2).

**Table 2:** Influence of CPSB21 inoculation on sunflower and tomato plant growth.

Treatment	Root length (cm)	Shoot length (cm)	Fresh weight (g plant^−1^)	Dry weight (g plant^−1^)	Chlorophyll (mg g^−1^ FW)	Total soluble proteins (mg g^−1^ FW)
**Sunflower**						
T0	20.1 ± 2.4ab	38.0 ± 4.1ab	31.24 ± 2.3a	4.32 ± 0.2ab	1.171 ± 0.09a	26.80 ± 1.6a
T1	17.0 ± 2.3abc	31.4 ± 3.0bcd	24.37 ± 2.4bc	3.68 ± 0.3bcd	0.729 ± 0.09bc	15.17 ± 1.2d
T2	14.7 ± 2.4bc	27.0 ± 2.6 cd	21.38 ± 2.1bc	3.24 ± 0.3 cd	0.644 ± 0.07bc	14.37 ± 0.9d
T3	12.9 ± 1.5c	24.8 ± 3.2d	19.12 ± 2.3c	2.89 ± 0.2d	0.557 ± 0.06c	13.40 ± 0.6d
T4	22.8 ± 2.1a	44.6 ± 2.7a	31.73 ± 2.4a	4.71 ± 0.2a	0.884 ± 0.07b	22.13 ± 1.5b
T5	18.9 ± 4.1abc	36.9 ± 2.3b	26.88 ± 2.4ab	3.82 ± 0.3bc	0.786 ± 0.07b	20.20 ± 2.0bc
T6	16.2 ± 1.4abc	32.8 ± 1.9bc	23.69 ± 3.1bc	3.27 ± 0.3 cd	0.680 ± 0.05bc	18.58 ± 1.2c
**Tomato**						
T0	14.0 ± 1.5a	33.0 ± 3.1a	27.31 ± 2.6a	3.53 ± 0.3bc	1.324 ± 0.07a	23.66 ± 1.5a
T1	11.8 ± 1.4abc	25.8 ± 2.7bc	20.45 ± 2.9ab	3.08 ± 0.2abc	0.795 ± 0.07bc	13.41 ± 1.8c
T2	10.1 ± 2.0bc	21.9 ± 2.1 cd	17.86 ± 2.6b	2.83 ± 0.1bc	0.702 ± 0.05 cd	11.97 ± 1.5c
T3	8.7 ± 0.7c	19.8 ± 1.2d	15.89 ± 3.2b	2.66 ± 0.1c	0.612 ± 0.07d	11.61 ± 1.3c
T4	13.4 ± 1.3ab	33.4 ± 1.9a	26.75 ± 3.6a	3.57 ± 0.2a	0.954 ± 0.06b	18.69 ± 1.6b
T5	11.2 ± 0.7abc	28.2 ± 2.4ab	22.77 ± 2.9ab	3.28 ± 0.4abc	0.856 ± 0.04bc	14.58 ± 1.2c
T6	10.9 ± 1.3abc	25.1 ± 1.0bcd	19.84 ± 2.3ab	3.01 ± 0.1abc	0.751 ± 0.07 cd	13.88 ± 1.5c

Values with different alphabets are significantly different from each other according to post hoc Tukey’s HSD (*P* < 0.05). Each value is a mean of three replicates.

To determine the biochemical response of strain CPSB21 inoculation on sunflower and tomato plants, total chlorophyll, and total soluble proteins were determined. Substantial changes in the total chlorophyll content in the leaves of sunflower and tomato plants were observed with CPSB21 inoculation as compared to control under Cr^6+^ stress. The effectiveness of the strain CPSB21 on the enhancement of chlorophyll content and total soluble proteins in sunflower and tomato is described in Table 2.

**Figure 3:**
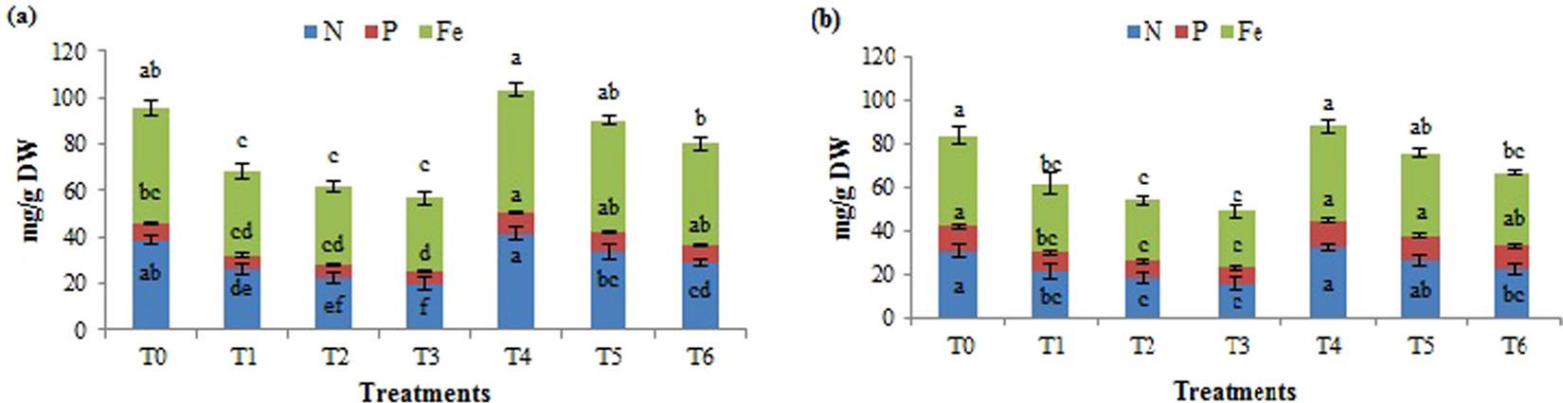
Nutrient uptake and Fe accumulation in plants (**a**) sunflower (**b**) tomato. Values with different alphabets are significantly different from each other according to post hoc Tukey’s HSD (*P* < 0.05). Each value is a mean of three replicates.

Data presented in Fig. 3 for N and P uptake and Fe (as micronutrient) accumulation shows that imposition of Cr^6+^ stress (T1, T2, and T3) substantially reduced the uptake of the three nutrients. However, inoculation with CPSB21 (T4, T5, and T6) was found to be effective in reducing the adverse effects of Cr^6+^ stress on N and P uptake and Fe accumulation.

**Figure 4:**
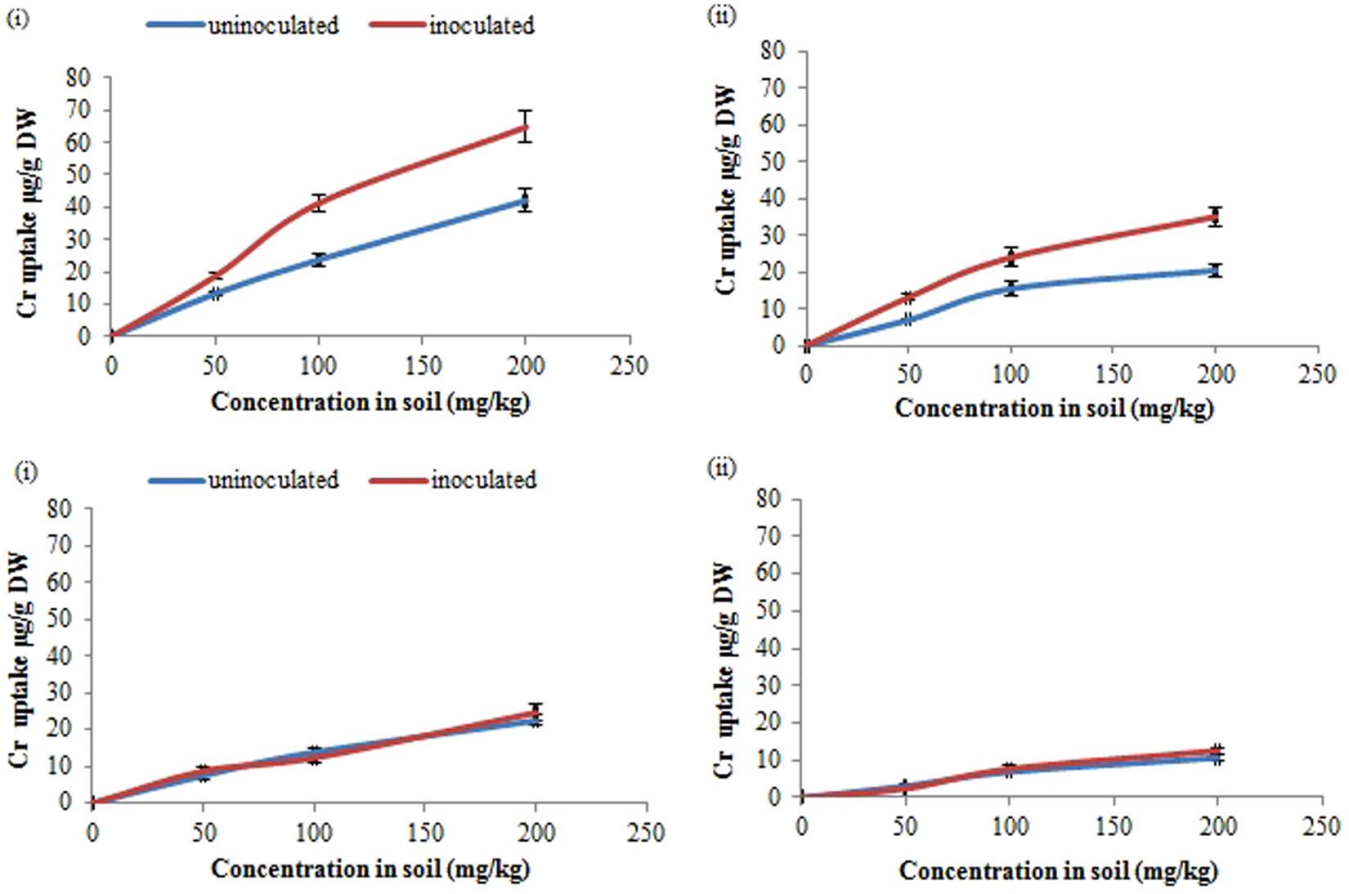
Chromium uptake (**a**) Sunflower: roots (i), shoots (ii); (**b**) Tomato: roots (i), shoots (ii) at 90 days of treatment with strain CPSB21 in sunflower plant.

Accumulation of Cr^6+^ in roots and shoots at 90 days of treatment increased with increase in dose of Cr^6+^ added to the soil. An increase in Cr^6+^ accumulation in sunflower was observed with CPSB21 inoculation (Fig. 4a). Also, in this study, no significant enhancement in Cr^6+^ uptake with CPSB21 inoculation was observed in tomato plant as compared to control (Fig. 4b). Moreover, inoculated and un-inoculated root system exhibited greater Cr^6+^ accumulation than the shoots.

### Antioxidant enzyme activities

The SOD activity in sunflower was increased with increase in Cr^6+^ dose (up to 100 mg L^−1^), then it maintained nearly constant, while in tomato an increase in SOD activity was observed with increase in Cr^6+^ concentration as compared to control. However, inoculation with strain CPSB21 further increased SOD activity in both the plants.

In sunflower, CAT activity was first increased with increase in Cr^6+^ concentration, and then it decreased, while in tomato plant, a gradual decrease in CAT activity was observed with increase in Cr^6+^ concentration. However, a significant enhancement in CAT activity was observed in both the plants with strain CPSB21 inoculation.

Under Cr^6+^ stress, an increase in GPOD activity was observed with increase in Cr^6+^ concentration in sunflower, while in tomato it first increased, and then decreased. However, the slight increase in GPOD activity was observed with strain CPSB21 inoculation.

### Lipid peroxidation

The effect of un-inoculated and CPSB21 inoculated Cr^6+^ treatments on MDA content is shown in Table 3. Compared to the control (T0), MDA content was increased with increase in Cr^6+^ concentrations (T1, T2, and T3 treatments) in both plants, indicating a rise in lipid peroxidation with increase in Cr^6+^ concentration. However, the MDA content of plants grown in Cr^6+^ contaminated soil inoculated with CPSB21 was lower than in un-inoculated soil. Low MDA content in plants is associated with less lipid peroxidation and consequently, decreased oxidative damage.

**Table 3:** Antioxidant enzyme activities upon CPSB21 inoculation.

Treatments	SOD (U mg^−1^ protein)	CAT (U mg^−1^ protein)	POD (U mg^−1^ protein)	MDA (nmol g^−1^ FW)
**Sunflower**				
T0	79.3 ± 3c	44.8 ± 2d	24.33 ± 5c	8.82 ± 1d
T1	163.3 ± 5b	53.4 ± 3 cd	29.48 ± 2bc	34.91 ± 2b
T2	181.8 ± 7ab	62.8 ± 4bc	46.85 ± 4a	42.22 ± 4a
T3	187.5 ± 8a	59.1 ± 4 cd	48.17 ± 5a	47.63 ± 2a
T4	185.8 ± 11a	75.4 ± 5ab	34.96 ± 3b	25.21 ± 2c
T5	193.0 ± 8a	83.6 ± 10a	53.63 ± 4a	30.10 ± 3bc
T6	205.0 ± 12a	77.8 ± 6a	53.85 ± 3a	33.05 ± 2b
**Tomato**				
T0	56.8 ± 4e	35.6 ± 3.4b	38.51 ± 2e	8.52 ± 1.6d
T1	131.8 ± 5d	32.5 ± 3.4bc	74.28 ± 3d	21.22 ± 2.4b
T2	158.1 ± 7c	26.9 ± 3.1 cd	82.64 ± 4bc	29.37 ± 2.9a
T3	180.2 ± 7ab	20.2 ± 2.8d	79.81 ± 5 cd	33.88 ± 2.9a
T4	150.6 ± 8c	47.2 ± 3.9a	83.71 ± 4bc	14.85 ± 2.8c
T5	176.2 ± 8b	38.7 ± 2.3b	92.80 ± 4a	19.98 ± 2.4bc
T6	194.7 ± 7a	30.5 ± 3.1bc	87.39 ± 3ab	22.49 ± 1.7b

Values with different alphabets are significantly different from each other according to post hoc Tukey’s HSD (*P* < 0.05). Each value is a mean of three replicates.

### Colonization of CPSB21

The ability of CPSB21 to colonize the rhizosphere of sunflower and tomato plants was tested after 90 days of inoculation. The survival rate of CPSB21 was greater in sunflower than in tomato rhizosphere. The cfu g^−1^ count in sunflower rhizosphere was 7.40 × 10^5^ (50 mg kg^−1^ treatment), 2.80 × 10^4^ (100 mg kg^−1^ treatment), and 4.90 × 10^3^ (200 mg kg^−1^ treatment), while in tomato the cfu g^−1^ count was 5.35 × 10^4^ (50 mg kg^−1^ treatment), 3.12 × 10^3^ (100 mg kg^−1^ treatment), and 1.6 × 10^3^ (200 mg kg^−1^ treatment).

### Characterization of the isolate CPSB21

**Figure 5:**
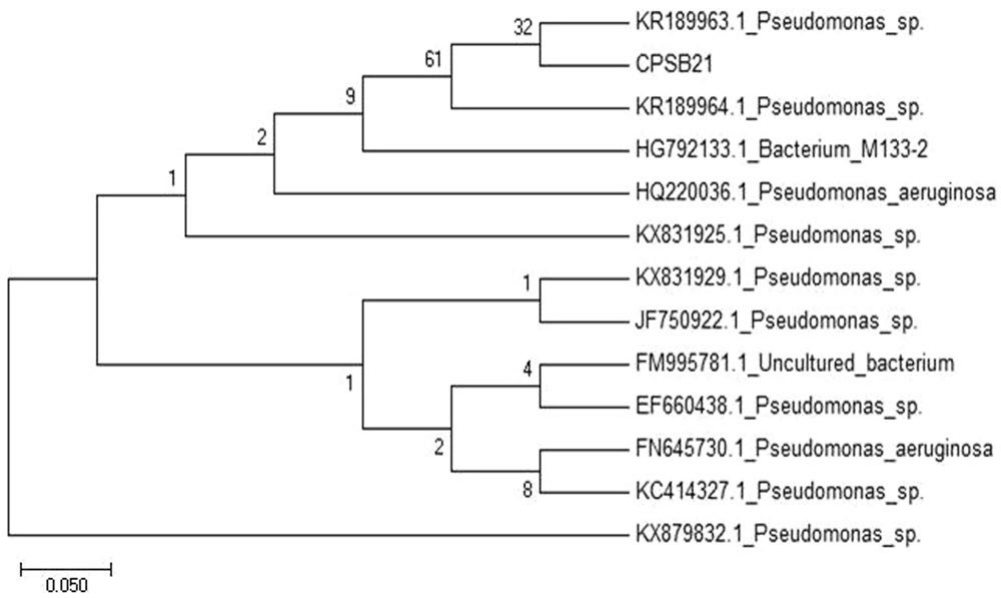
Phylogenetic analysis of the isolate CPSB21 constructed using 16S rRNA gene sequence applying neighbor joining method.

The Cr^6+^ resistant PGP bacterial strain, CPSB21, was tentatively identified as *Pseudomonas* sp. on the basis of biochemical and morphological characterization (SM2). Analysis of 16S rRNA gene sequence using BLASTn program at NCBI showed similarity to *Pseudomonas* sp. The phylogenetic tree of the isolate CPSB21 is shown in Fig. 5. The nucleotide sequence of the strain CPSB21 is deposited in NCBI database with accession number - **MG333693.1**

## Discussion

In the present study, isolate CPSB21 exhibited maximum P solubilization, however the solubilization decreased with increase in Cr^6+^ concentration. This effect may be due to the toxicity of Cr^6+^ on the test strains. The decrease in P solubilization by *Pseudomonas aeruginosa* with increase in Cr^6+^ stress had also been reported^19,20^. Siderophores production was also decreased with increase in Cr^6+^ stress. This reduction in siderophores under metal stress may be attributed to the destruction of microbial membrane bound ferric reductase enzymes. Several studies had reported the potential of microorganisms in siderophores production^21,22^. In addition to PGP potential, metal resistant-PGP bacteria also possess certain traits (acidification, production of iron chelators, siderophores, and organic acids) that can alter the heavy metal mobility and availability to the plants^23^. The results of the present study disclose that isolate CPSB21 facilitated the release of Cr from the soil. These findings suggest that the Cr^6+^ resistant PGP *Pseudomonas* sp. CPSB21 may influence the mobilization of Cr from soil and renders an efficient phytoremediation process.

In the present study, an increase in plant growth parameters was observed with CPSB21 inoculation. This increase in plant growth by CPSB21 under Cr^6+^ stress in soil may be attributed to its ability to produce siderophores, IAA, NH_3_, HCN, and P solubilization^24^. Similar to our results, bacterial mediated plant growth in sunflower and tomato under stress conditions has been reported^25,26^. Moreover, the inoculation of CPSB21 enhanced the chlorophyll content in both the plants. Our results are completely in agreement with the previous study^27^ which reported that under stress conditions the metal resistant PGP inoculation could activate the defense mechanism in plants that reverses the loss of chlorophyll content in plants. The bacterial inoculation under stress enables the plant to synthesize more chlorophyll by providing additional nitrogen and iron sources^28^.

Cr stress also affects the nutrient uptake by plants in a complex manner. Cr competes with Fe binding sites, hence interferes with Fe absorption in plants. This leads to decrease in Fe accumulation required for chlorophyll and heme biosynthesis^29^. However, inoculation of bacteria improves the nutritional status in plants by an unknown mechanism^30^. In our study, the inoculation of CPSB21 enhanced the uptake of N and P and increased the Fe accumulation under stress. The increase in Fe accumulation by plants upon inoculation might be due to siderophores production by the inoculant CPSB21 that enhanced the availability of Fe to plants under stress^31^.

An increase in Cr^6+^ uptake by sunflower was observed with strain CPSB21 inoculation. In contrast to our study, decrease in chromium accumulation on inoculation of *Bacillus* sp. in chickpea was reported previously^32^. This variation in the present study might be due to the difference in plant species, uncontrolled environmental conditions, and plant associated factors such as root exudates, and root associated processes^33^. However, inoculation of the strain CPSB21 has not shown any positive results in response to Cr^6+^ uptake in tomato. These results were concurrent with the previous findings^34^ which has recorded similar observations upon inoculation of *Pseudomonas* sp. under Cr^6+^ stress in maize plant. Also, greater Cr^6+^ accumulation was observed in roots than shoots in both plants. This may be attributed to the poor translocation of chromium from the roots to the shoot system^35^.

Plants employ detoxifying antioxidative system to maintain ROS at an optimum level. The exposure to Cr^6+^ stress causes ROS production, hence resulting in high oxidative damage. The antioxidative enzymes include SOD, CAT, and POD. SOD is responsible for converting superoxide into H_2_O_2_, while CAT and GPOD are basically involved in the dismutation of H_2_O_2_ to H_2_O and O_2_. However, antioxidant enzyme activities in metal-stressed plants are highly variable, depending on the concentration of metal, plant species, exposure duration, and metal ion^36^. In response to heavy metal stress, SOD activity shows the biphasic response, it may induce with increase in concentration of heavy metals or may increase at a low metal dose further becomes constant with increase in metal concentration^37^. Increase in CAT activity in response to metal stress is observed in many plants^38^, and an increase in CAT activity is supposed to be an adaptive trait^39^. However, in few studies the decrease in CAT activity with increase in metal concentration is also observed in some plants^40,41^. The enhanced antioxidant enzyme activity with CPSB21 inoculation in the present study might be due to the increased gene/mRNA expression of plant antioxidant enzymes than un-inoculated plants^15^.

MDA formation is a result of lipid peroxidation that can react with free amino-group of protein, causing intra-molecular, and intermolecular cross linking of proteins, thus resulting in cell damage^42^. In the present study, higher MDA was noticed in Cr treated plants (T1, T2, and T3), which may be due to the imbalance between the production and removal of free radical in cells^43^. The decrease in lipid peroxidation with CPSB21 inoculation under stress may be due to the enhanced production of ROS scavenging enzymes. The bacterial inoculation activates the gene expression profile of metal detoxifying enzymes to cope up the metal stress^44^.

## Conclusion

A rhizospheric bacteria, *Pseudomonas* sp. (strain CPSB21), isolated from the tannery effluent contaminated agricultural soil was Cr^6+^ resistant and exhibited PGP traits. Inoculation of the isolate CPSB21 to sunflower and tomato enhanced the plant growth, antioxidant enzyme activities and reduced lipid peroxidation. The strain CPSB21 also enhanced the uptake of Cr^6+^ in sunflower which suggests the potential of the strain in the enhanced phytoremediation process. The findings indicate the potential of the strain CPSB21 for bioremediation of Cr^6+^ contaminated agricultural soils.

## Methods

### Analysis of soil samples

Rhizospheric soil samples were collected from the Cr contaminated agricultural fields nearby tannery industrial area in Jajmau, Kanpur, India. Collected samples were air dried, sieved, and kept for the analysis. A part of the samples was kept in dark at 4 °C for microbial studies. Soil pH and electrical conductivity (EC) were determined by soil: water (1:2.5 w/v) suspension. Organic carbon (OC) was determined by rapid dichromate oxidation technique^45^, available nitrogen (Av. N) by alkaline permanganate method^46^, available phosphorus (Av. P)^47^, and available potassium (Av. K) by ammonium acetate extraction method^48^. The Cr^6+^ concentration was determined as per US EPA 3060a^49^.

### Isolation of Cr^6+^ resistant strains

The Cr^6+^ resistant strains were isolated by serial dilution and pour plate methods using Sucrose Low Phosphate (SLP) agar media (sucrose-1%, (NH_4_)_2_SO_4_ - 0.1%, K_2_HPO_4_ - 0.05%, MgSO_4_ - 0.05%, NaCl - 0.01%, yeast extract - 0.05%, pH - 7.2) amended with 50 mg L^−1^ Cr^6+^. This media was designed to avoid metal salt precipitation. The plates were incubated at 30 °C for 5 days. The isolates were tested for minimum inhibitory concentration (MIC) by gradual increase in Cr^6+^ (50–1200 mg L^−1^) amended on SLP plates over 5 days of incubation.

### PGP traits of the isolates under Cr^6+^ stress

The phosphate solubilization potential of the bacterial isolates was tested using Pikovskaya’s broth^50^. The IAA production was quantitatively analyzed using Salkowski’s reagent^51^ with 0.2% tryptophan. Siderophores secretion by the isolated strains was tested qualitatively using blue agar^52^ (Himedia, India) containing CAS dye with graded Cr^6+^ concentrations. NH_3_ and HCN produced were evaluated as per the standard protocols^53,54^.

### Pot trials

Seeds of sunflower and tomato were used in this experiment, while the soil sample was collected from the agricultural field nearby tannery industrial area in Jajmau, Kanpur, India.

The procured seeds were surface sterilized using 70% ethanol followed by 3% hypochlorite solution for 3 min and were shade dried. Sterilized seeds were coated with isolated CPSB21 by dipping the seeds in culture broth (10^8^ cells mL^−1^) for 2 h using gum Arabic (10%) as an adhesive. The seeds dipped in sterile distilled water served as control. The treated and untreated seeds of sunflower and tomato (5 seeds per pot) were sown in clay pots containing 3 kg sterilized soil. The Cr^6+^ concentration in the pots was maintained to 50, 100, and 200 mg kg^−1^. The soil samples were left for two weeks for metal stabilization. On total seven treatments were set up in triplicate in a complete randomized design for both plants. The details of the treatments are: T0 (control, uncontaminated soil), T1 (soil- 50 mg kg^−1^), T2 (soil- 100 mg kg^−1^), T3 (soil- 200 mg kg^−1^), T4 (soil- 50 mg kg^−1^ + CPSB21), T5 (soil- 100 mg kg^−1^ + CPSB21), T6 (soil- 200 mg kg^−1^ + CPSB21).

After germination, the plants were thinned to two per pot. The pots were kept in open conditions and watered with tap water daily. Plants were harvested after 90 days of seed sowing and roots were washed with deionized double distilled water and dried. Root and shoot were separated and biomass were recorded after oven drying (70 °C for 5 days). Chlorophyll content, nitrogen (N) and phosphorus (P), and iron (Fe) were determined as per the standard protocols^55–57^. For Cr^6+^ analysis, the plant samples were oven dried at 105 °C for two days (so as to attain constant weight), ashed in a muffle furnace at 600 °C for 6 h, and dissolved with a mixture of 2 M HCl and 1 M HNO_3_. The samples were filtered and final volume was made up to 50 mL^58^. The samples were further analyzed using 1, 5 - diphenylcarbazide. The absorbance was measured at 540 nm using spectrophotometer (UV - 1800, Shimadzu, Japan).

### Assay of antioxidant enzymes

For enzyme extraction, fresh leaf samples (0.5 g) were ground with mortar and pestle and homogenized in an ice cold potassium phosphate buffer (10 ml, pH 7.0). The homogenate was centrifuged at 4 °C for 20 min at 12,000 rpm. The resulting supernatant was stored at 4 °C for determination of various antioxidant enzymes. SOD activity was measured through the photoreduction of nitroblue tetrazolium chloride (NBT)^59^. The CAT and G-POD activity was determined as per the standard protocols^60,61^.

### Determination of lipid peroxidation

To evaluate the extent of Cr^6+^ induced oxidative damage to membranes, the changes in lipid peroxidation were measured by evaluation of MDA formation in the leaves of sunflower and tomato plants. Briefly, leaf samples (0.4 g) was homogenized in 0.1% trichloroacetic acid (6 ml) and centrifuged at 10000 RPM for 10 min. To the supernatant (1 ml), 20% trichlorocaetic acid (4 ml) containing 0.5% thiobarbituric acid was added. The mixture was heated at 95 °C for 30 min and the absorbance was taken at 532 nm and 600 nm^62^.

### Characterization of the isolate CPSB21

Morphological and biochemical identification tests of the isolated bacterial strain CPSB21 were carried out by the standard protocol outlined in Bergey’s Manual of Systemic Bacteriology. Molecular identification of the isolates was done using 16S rRNA gene sequencing. The primers 27 F (5′-AGAGTTTGATCMTGGCTCAG-3′) and 1492 R (3′-TACGGYTACCTTGTTACGACTT-5′) were used for amplification. PCR was performed using following thermal cycling conditions: denaturation (94 °C for 3 min), annealing (50 °C for 60 Sec), and extension (72 °C for 10 min). Sequencing reactions were performed using an ABI PRISM® BigDyeTM Terminator Cycle Sequencing Kits with AmpliTaq® DNA polymerase (FS enzyme) (Applied Biosystems).

Phylogenetic analysis was performed using the obtained sequence followed by the alignment with the NCBI nucleotide database. The closest species related to the sequence were retrieved and analyzed by MEGA software (version 7). The neighbor joining method was employed with bootstrap values generated from 1000 replicates.

### Data analysis

To determine the mean and standard deviation of the data sets, XLSTAT package of MS Excel 2010 was used. One way analysis of variance (ANOVA) followed by Tukey’s HSD at 5% (*P* < 0.05) probability level was performed between different treatments in pot scale study. All treatments were carried out in triplicates.

## Electronic supplementary material

Supplementary Information
