# Supplementary material for: Potential applications of Pseudomonas sp. (strain CPSB21) to ameliorate Cr6+ stress and phytoremediation of tannery effluent contaminated agricultural soils
Source: Sci Rep. 2018 Mar 20;8:4860. doi: 10.1038/s41598-018-23322-5 (PMC5861048; doi:10.1038/s41598-018-23322-5)

**Potential applications of *Pseudomonas* sp. (strain CPSB21) to ameliorate Cr^6+^ stress and phytoremediation of tannery effluent contaminated agricultural soils**

Pratishtha Gupta, Rupa Rani, Avantika Chandra, Vipin Kumar^*^

Department of Environmental Science and Engineering,

Indian Institute of Technology (ISM), Dhanbad-826 004, Jharkhand, India.

*Correspondence

Email: [vipinmicro1@gmail.com](mailto:vipinmicro1@gmail.com); Tel:  +91-9471191352 (M), +91-326-2235643 (O)

**SM1** Characteristics of soil used for the isolation of Cr resistant bacteria and pot trails

| Parameters | Value |
| --- | --- |
| pH | 7.4-7.9 |
| EC (mS cm^-1^) | 0.63-2.4 |
| OC (%) | 1.14-1.76 |
| Available P (mg kg^-1^) | 5.03-12.67 |
| Available N (mg kg^-1^) | 128.63-139.52 |
| Available K (mg kg^-1^) | 73.41-87.13 |
| Cr^6+^ (mg kg^-1^) | 18-30 |

**SM2** Morphological, Biochemical, and Physiological traits of the isolate CPSB21

| Morphology | Traits |
| --- | --- |
| Gram reaction  Shape  Colour | -  Rod  Yellow |
| Biochemical Tests | Traits |
| Indole  Methyl Red  Vogues-prokauer  Citrate utilization  H_2_S production  Oxidase  Nitrate utilization  Gelatin hydrolysis  Catalase | -  -  +  +  -  -  -  -  + |
| Carbohydrate Fermentation | Traits |
| Lactose  Sucrose  Mannitol  Glucose | +  +  +  + |

**SM3** Bland-Altman plot for different plant parameters (Tukey’s difference in means plot).


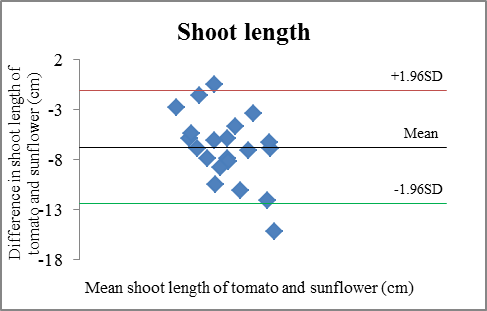

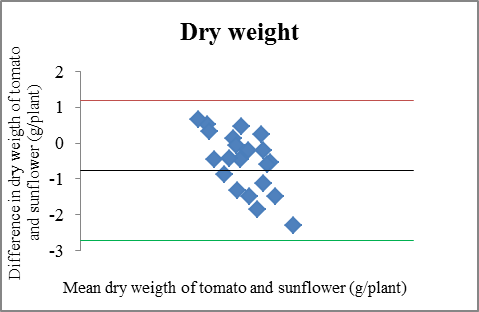


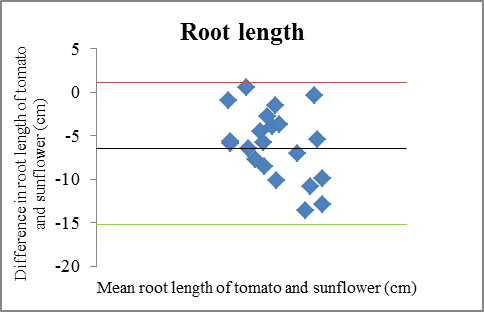

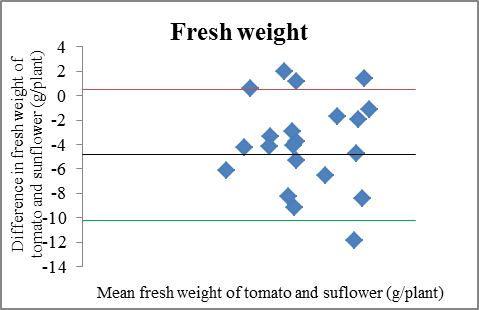


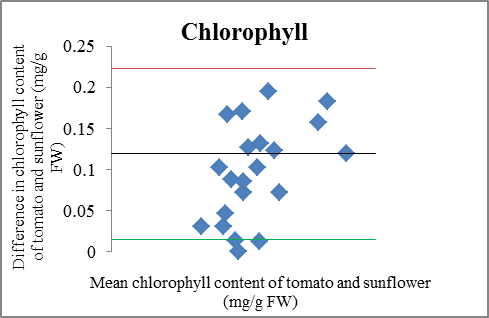

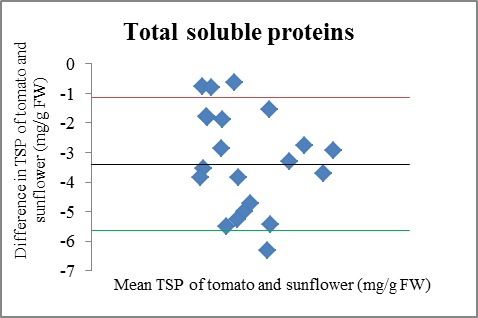


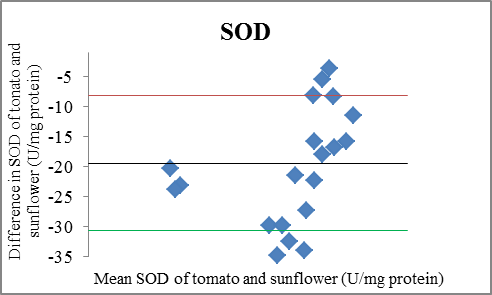

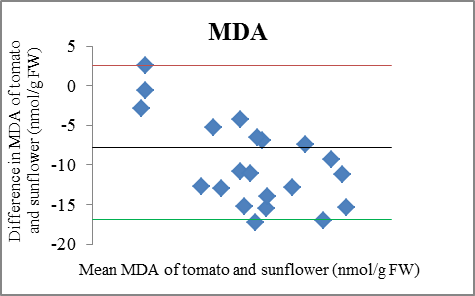


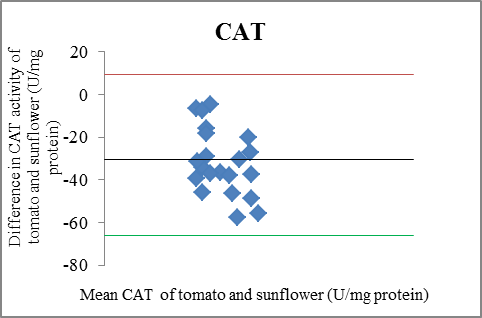

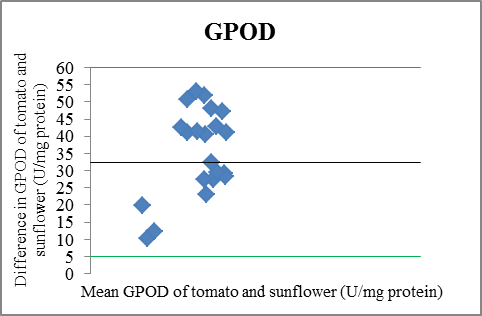

Supplement: Supplementary file 1 — Supplementary Information [file 41598_2018_23322_MOESM1_ESM.doc]
